# Substance Use of Older Adults in Gerontological Social Work Client Documents

**DOI:** 10.1177/14550725261419965

**Published:** 2026-02-19

**Authors:** Ilona Kaitala, Minna Zechner, Teija Karttunen, Eeva Rossi

**Affiliations:** 1 3835 Faculty of Social Sciences, University of Helsinki, Helsinki, Finland; 2 Faculty of Social Sciences, University of Lapland, Rovaniemi, Finland

**Keywords:** client documentation, gerontological social work, older adults, service assessment of needs, substance abuse, substance use

## Abstract

**Aims:**

This study describes the substance use of gerontological social work clients and the ways it affects their use and needs for social and healthcare services, as featured in client documents written in the context of assessment of the need for services.

**Methods:**

The data consist of gerontological social work client documents of 55 clients from one municipality in Finland. The data were analyzed using reflexive thematic analysis in two stages. The first stage addressed the question of documenting substance use and the second stage included clients with ongoing substance abuse (*N* = 15).

**Results:**

Substance use was common among the sampled gerontological social work clients. Only a small number of clients were identified as having harmful substance use, and these clients had complex needs for services and multiple uses of services. However, their service use and needs were rarely documented from the point of substance use, even though problematic substance use had been included in legislation as one criterion for having complex needs. Thus, some of the service professionals seemed to have ignored substance use when assessing the needs of older clients.

**Conclusions:**

There is need to develop social work training to increase the professional knowledge base and identify or bring up matters of substance use when encountering older clients with substance abuse.

## Introduction

Service need assessment within gerontological social work is a process where harmful substance use among older adults may be identified. Experiences of substance abuse^1^ is a growing social problem among older adults (Chhatre et al., 2017). Especially the frequent use of alcohol is rather common amongst older adults in Finland and in other Nordic countries. Denmark has the highest prevalence of frequent older drinkers, followed by Finland, Sweden and Norway, in that order (Tigerstedt et al., 2020).

Because social work professionals have frequent contacts with clients, they have the opportunity to identify and intervene in substance abuse (Wadd & Galvani, 2014). The aim of gerontological social work is to ensure and enhance the well-being and quality of life of older adults (aged 65 years and over) and their families. Additionally, the aim is to support older adults’ capabilities and resolve social problems that impact their everyday lives and to make visible the structural inequalities and disadvantages encountered in older age (Ray et al., 2015; Tiilikainen et al., 2025). In Finland, older adults with substance abuse issues are more likely to use social services than specialized substance use services. There are very few substance abuse services targeted for older adults, which means that older adults have difficulties in accessing these services (Kuussaari et al., 2021).

The present study focuses on substance use among older adults in the context of gerontological social work in Finland, using client documents from service needs assessment as data. Social work documents are central when assessing the needs of older adults, and when determining, evaluating and describing their circumstances (Olaison, 2010). The core task of service needs assessment is different from a medical assessment of substance abuse, with the latter aiming to detect, identify and assess problematic substance abuse, and, when necessary, to make a diagnosis of substance use disorder. However, making substance use disorder diagnoses is not within the scope of practice of social work professionals. Therefore, in the present study, we use the definition of “substance abuse” to refer to the harmful use of alcohol and the misuse of prescribed drugs, both of which have negative health and social impacts for social work clients (Lundgren & Krull, 2018: p. 10).

Although a social worker does not need to be an expert in substance abuse problems, they still need the confidence to discuss and explore the issues of substance abuse with clients. However, social and healthcare professionals tend to have difficulties in recognizing or addressing problematic substance use among older adults (Bareham et al., 2020; Weber et al., 2022). This relates to the professionals’ negative assumptions and false beliefs identified in studies; for example, the baseline assumption that older adults’ drinking behavior is impossible to change, or that older adults do not abuse alcohol or drugs (John Henley et al., 2018; Wadd & Galvani, 2014). Substance use may also be a difficult topic to discuss for professionals due to their perceptions of substance use being a sensitive topic that interferes with clients’ rights to self-determination and privacy, or as a topic that clients are unwilling to discuss (Bareham et al., 2021; Johannessen et al., 2021). Similar to the professionals, older adults themselves may have difficulties in identifying their substance abuse; for example, they may have challenges in estimating the amount of alcohol they consume. Additionally, older adults may be ashamed of substance use, which prevents them from seeking help (Wadd & Galvani, 2014).

In general, there is limited research on older adults’ substance use related to the use of social and healthcare services (Bach et al., 2024; Kuussaari et al., 2021) and only a few studies of older adults’ substance abuse (Rosen et al., 2018), client documentation (Niemi & Pietilä, 2023; Olaison, 2010) and substance use connected to assessment (John Henley et al., 2018) have been conducted in the context of gerontological social work.

The aim of the present study is to address the problematic substance use of older adults and its impact on their service use and service needs by analyzing gerontological social work client documentation of social work and social care professionals. The broader purpose is to portray the addressing of substance use of older adults in the social service system. The research questions, asked in the context of gerontological social work client assessment documents, are: (1) How is substance use visible in the documents? (2) What client service needs are connected with substance abuse? (3) What service use is connected with substance abuse? The study context is Finland, which has a strong (Nordic) welfare state model meaning that the state has the overall responsibility for providing care and social work for older adults (Olaison, 2017; Tiilikainen et al., 2025). At the time of the data collection in 2021, similar to other public social and healthcare services, gerontological social work was provided by municipalities. Since 2023, those services have been organized by 21 wellbeing service counties and the city of Helsinki.

### Needs assessment and client documents

Assessment is at the essence of professional social work practice (Geron, 2006) and central in the allocation of social and health services. Assessment definitions vary in social work literature (Hepworth et al., 2013; Milner et al., 2020). Assessment is a process that occurs between the social worker and the client, and includes gathering, analyzing, synthesizing and interpreting information to form a conclusive statement and picture of the client's situation, needs, and strengths (Hepworth et al., 2013). Dyke (2019: p. 18) describes assessments as an “exercise in professional judgement”. The lives of clients should be understood as a combination of contextual and environmental factors (Clifford, 2018), and Milner et al. (2020) have structured assessment as a five-stage process, including preparing, collecting data, applying professional knowledge, making judgements, and deciding and/or recommending.

Assessment can be considered as an ongoing process (Minimol, 2016), although institutional policies and practices may tie it specifically to the intake phase of the social work process, as is the case in this study. In assessing older adults, the client's strengths-based, bio-psychosocial, and life course or life history perspectives go beyond the risk-oriented, deficit approach and medical model. A comprehensive approach in social work assessment in older adults aims to develop the internal and external resources of clients, to maintain activities and opportunities, and to mobilize their capabilities (Clifford, 2018; McInnis-Dittrich, 2015; Minimol, 2016).

McInnis-Dittrich (2015) promotes biopsychosocial assessment with older adults and has presented components for a comprehensive assessment including issues of physical health, competences in activities of daily living, psycho-emotional functioning, social functioning, religion and spirituality, environmental safety, and sexuality. McInnis-Dittrich (2015) further suggests that assessment with older adults is both a process and a product, where the process implies forming and maintaining trust and a relationship between the social worker and the client, and the product is the care plan which is continuously monitored together.

In Finland, care managers, who are health or social care professionals, assess the needs of older adults and eligibility for different health and social services, including social work (Kinni & Tiilikainen, 2024; Peiponen et al., 2023). The legal foundations for their work are found in the Act Supporting the Functional Capacity of the Older Population and on Social and Health Care Services for Older Persons (980/2012) and the Social Welfare Act (1301/2014). The Social Welfare Act 36 stipulates that if older adults have complex needs, they must be assessed by a qualified social worker.

Older social work clients with complex needs have different kinds of functional decline, social challenges and problems such as domestic or substance abuse (Ray et al., 2015; Soukiala & Pietilä, 2024). In addition, the concept of “complex needs” refers to older adults’ problematic life situations in which multidimensional, long-term, and cumulative disadvantages are combined (Rossi et al., 2025; Tiilikainen et al., 2025). Such needs and situations require gerontological social work interventions and long-standing work with clients. Furthermore, the Social Welfare Act includes a category of clients in need of special support, which can be interpreted as referring to people with complex needs. Importantly, it has been noted that this act especially mentions people with harmful substance use as a typical group included in this category (Kuusisto et al., 2021).

Social work in Finland has had a central or even leading professional position in substance abuse treatment (Kuusisto & Ranta, 2020). This has changed as a result of changes in national legislation and policy programs, and by way of medicalization. It means that healthcare professionals play a key role in conceptualizing, assessing, and treating substance abuse disorders (Health Care Act, 1326/2010; Santala, 2022). The use of assessment instruments such as AUDIT (i.e. Alcohol Use Disorder Test) or methods such as SBIRT (i.e. Screening, Brief Intervention and Referral to Treatment) or brief intervention (Schonfeld et al., 2015) is not common in assessment in social services in Finland. However, the use of these is recommended (Renko, 2016) and there are also assessment instruments especially for older adults; for example, the SAMI (i.e. Senior Alcohol Misuse Indicator) tool or SMAST-G (i.e. Short Michigan Alcoholism Screening Test-Geriatric Version) (SAMHSA, 2020: p. 45). The Finnish acts steering assessment are framework legislation without detailed instructions for needs assessment, apart from the standardized tool Resident Assessment Instrument (RAI), which has been obligatory since 2023 when working with older adults (Kinni & Tiilikainen, 2024). RAI also includes questions about substance use (Finnish institute for Health and Welfare, 2025).

The role of needs assessment is crucial (Kinni & Tiilikainen, 2024) because care managers and social workers are decision-making authorities whose decisions about older adults’ access to social care services should be based on the needs of the clients (Tynkkynen et al., 2022). It is further noted that access to social services is influenced by both the resources of the service system and the competencies of the professionals assessing clients’ needs (Peiponen et al., 2023). This also underlines the dependence of decision-making authorities on legislation and local guidelines (Wittberg, 2024), even though, in Finland, public officials have a certain amount of discretion power and are responsible for making decisions about older adults’ access to services.

An important part of the assessment is documentation, and its results in the form of client documents are used here as data. Documentation in social work can be defined as an institutional activity and as a personal professional work process. Here, social welfare professionals are making choices while writing the document, but the produced documentation may be affected by work-related guidelines and restrictions (Kääriäinen, 2022). Consequently, when analyzing client documents, it must be considered that these documents reflect the perspectives of social welfare professionals, the institutional practices of the social welfare system and the client documentation systems, which usually steer to ask about substance use. Social work client documents are an instrument for producing and passing on information, and they reflect the prevailing practices of working with and care for older adults (Olaison, 2010). At the individual level, assessment documents constitute a foundation for identifying and making decisions regarding service planning and delivery for clients (Foster et al., 2008).

## Methods

The data for the present study consist of gerontological social work client documents of 55 clients from one municipality, collected in 2021 in a research project (‘Gerontological social work responding to complex needs of older adults’ (VN/25316/2020) ). The selection criteria were defined by researchers and applied by social workers. They included clients that had complex needs according to the Social Welfare Act, and that they had been a client for a long period of time, or that there were multiple documents per client. In the data, the clientship for gerontological social work commenced between 2015 and 2021, and almost one-third of the clients had previously been clients of adult social work. Client documents offer a specific professional-dominated view into the life situations of individuals who are unlikely participants in research. These documents represent both the institutional and legal rules that govern documentation and the choices made by employees while doing the documentation (Niemi & Pietilä, 2023). Analyzing client documents thus opens a professional-led view towards older adults with substance abuse issues.

Of the 55 clients, 35 were women and 20 men, and they were born between 1927 and 1955. The clients had four different types of client documents, including service needs assessment, care and service plans, situation assessments, and statements from professionals. The data comprised a total estimated number of 2,033 pages adding up to 813,086 words. For this study, we selected those documents that were related to needs assessment and the assessment of the situation, which are part of the key processes of identifying substance abuse in older clients. Even though the documents are taken from gerontological social work, the texts have been written by a variety of social and healthcare professionals, including social workers, social counselors, client managers and nurses. When professionals document client data, they choose the concepts they use and what they write. These choices are based on their education, professional practices and work positions. Thus, healthcare professionals predominantly focus on issues related to health, when social work professionals generally have a broader perspective. Even though we approached the data as an entity, not focusing on differences between the documentation of different professionals, the differences in professionals’ emphases were visible in some instances. However, the client documentation system and organizational rules create some shared foundation for the documentation.

The data used in this study are sensitive, and the clients of gerontological social work can be understood as a vulnerable group. Accordingly, the research subjects’ intactness must be considered in the research process (Nikander & Zechner, 2006; Rauhala & Virokannas, 2011). We have been careful to follow the Finnish Code of Conduct for Research Integrity (The Finnish National Board on Research Integrity, 2023) and General Data Protection Regulation (GDPR) (European Parliament and Council, 2016) and appropriate research permission has been obtained from the focus municipality. The data have been carefully anonymized, and we have taken care to write respectfully about the clients and social work professionals and avoid expressions that might conceptually stigmatize or damage the research subjects involved.

The data analysis was reflexive thematic analysis, which is a theoretically flexible analysis method and refers to a practice of critical reflection on the researcher's role in the process (Braun & Clarke, 2022: p. 5 & 9). In this study, the researchers’ reflexive role was especially related to making interpretations drawn from the client documentation and ethical considerations relating to the use of the data. The researchers had to interpret the data from a perspective of substance use and reflect over the documentation focusing on clients in a vulnerable position. From an ontological stance, the analysis was committed to realism, which assumes that the data present a certain reality which, in the present study, is the professionals’ interpretation on the clients’ situations. The purpose is to represent the content of the data accurately and objectively (Braun & Clarke, 2022: p. 168). Consequently, the focus is placed on capturing professionals’ perceptions of the phenomena; in this case, how the reality is construed in the client documentation, which is simultaneously influenced by restrictions of client information systems and the professional settings. However, it is important to note that criticism has previously been directed towards social work documentation as being inadequate and lacking a client view (Kuorikoski, 2024), which can lead to one-dimensional descriptions on the clients’ situations based solely on the interpretations of the professionals. This should be taken into consideration when evaluating our results.

The analysis proceeded by adapting a six-phase model of thematic analysis. First, the first author read the dataset twice to familiarize themselves with the content while keeping account of the research questions under study. The first investigator (IK) then read the entire data systematically and tagged segments that were potentially relevant to the research questions, with the purpose of demarcating differences and shared or similar meanings and then coding the identified groups. The coding process was inductive (data-driven) and relied on latent coding (research-driven) which focuses on deep and implicit meanings. (Braun & Clarke, 2022: pp. 35–58).

In the third phase, similar codes were clustered into themes, and the initial meaning patterns were explored. The fourth phase included assessing and reviewing the themes and comparing them to the original codes. In the fifth phase, each theme was labeled with an informative name. The sixth phase (writing) was already commenced in the third phase with the purpose of identifying the analysis process as it formed in the writing examination (Braun & Clarke, 2022: pp. 35, 36 & 79). The analysis was conducted using the ATLAS ti-program (https://atlasti.com).

The analysis was conducted in two stages, based on the research questions. Both stages included the six phases of thematic analysis identified above. The first stage addressed the question of documenting substance use, and all 55 clients were included. The analysis resulted in the first theme (I) titled “Varying levels of substance use”. This theme included five code groups: (1) clients whose substance use is unclear; (2) clients not using substances; (3) occasional or minor use of alcohol; (4) heavy use of alcohol or abuse of prescription drugs; and (5) clients who have background of heavy alcohol use.

After reviewing the documents of 55 clients, it became evident that ongoing substance use that affected the life of the client was present in some way in the documents of 15 clients, with these clients belonging to code croup (4). This client group consisted of seven women and eight men, and these clients had an average of three or four documents, varying from one to nine documents. Hence, the documents of these 15 clients were selected for the second stage of analysis. In this stage, the aim was to answer questions regarding the use and need for services of those older adults with substance use issues. The analysis resulted in three themes (II, III and IV). The second theme II was named “Complex needs for services”. Theme III was named “Diverse support network” and the last theme was named “Challenges in accessing social and healthcare services”. All themes included code groups that are presented in Table 1. The results of the analysis are presented in Table 1.

**Table 1.** The results of the analysis: themes I–IV and the included group codes.

Themes			
(I) Varying levels of substance use	(II) Complex needs for services	(III) Diverse support network	(IV) Challenges in accessing social and healthcare services
Code groups			
Clients whose substance use is unclear	Weak functional capacity and health condition	Substance use related services	Clients’ negative or ambivalent attitudes towards services
Clients not using substances	Financial support and coordination of other services	Other services	Reasons independent from the client
Occasional or minor use of alcohol	Substance use- related needs	Support from relatives and other close persons	
Heavy use of alcohol or abuse of prescription drugs	Daily functions		
Clients who have a background of heavy alcohol use	Abuse in close relationships		

## Results

Next, we present the four themes emerging from the data analysis. It is essential to keep in mind that, even though we present the results in a factual manner, the data consist of client documents that provide a specific professional-provided and gerontological social work-based view of substance use among older adults and need use, as well as the challenges related to services.

### Theme I: Varying levels of substance use

Although present substance use appeared to be common amongst the clients, over one-third (*N* = 20) of the clients did not seem to use any substances at all. The documents also included mentions of a client's substance use as being unknown or unclear, and, at times, substance use was not discussed with the client: “We did not discuss the topic during the client meeting” (F-1934).^2^Research (e.g., Bareham et al., 2020; Johannessen et al., 2021) indicates that social and healthcare professionals have challenges in identifying or discussing substance use with older clients. Some of these clients used alcohol rarely or in small amounts: “The client said that they use alcohol moderately, once a week, and without binge-drinking …” (F-1950). Professionals did not portray these clients’ alcohol use as being harmful or having negative effects on their lives. Rather, moderate and non-harmful alcohol use was presented as a part of clients’ everyday life and daily routines; for example, having small amounts of alcohol with dinner, or a weekly drink after a sauna. Moderate and non-harmful alcohol use was also often related to social gatherings or festive occasions: “(The client) does not use alcohol harmfully. Might take a glass of wine every now and then at a party” (F-1927). Thus, alcohol use which was considered moderate was mainly defined as being an integral part of social encounters (Emiliussen et al., 2021; Karlsson & Gunnarsson, 2018; Kuerbis, 2019).

Problematic substance use is one of the factors which is used to define clients with complex needs (Kuusisto et al., 2021), although, in the present study, only a small portion of all clients were identified as having harmful alcohol use or misusing prescribed drugs. In a previous study, social work professionals most often recognized clients with substance use as having a need for special support. The professionals’ interpretations about substance use as a condition meriting special support emphasized the client's long-term addiction, regular substance use, and the use of drugs (Kuusisto et al., 2021). In the present study, the clients who were described as having harmful substance use used alcohol frequently and in large amounts: “The client says that they use alcohol almost daily, and three or four times a week they drink spirits” M-1949). In this category, professionals assessed the clients’ uses of alcohol as being harmful to the client themselves, describing it as heavy, hazardous, or a matter of concern with negative consequences: “Heavy alcohol use has made the client ill” (F-1952). Even though the professionals saw these clients’ alcohol use as harmful, some clients were described as denying the negative effects of their substance use: “The client explains that they drink for relaxation or in order to feel good. The client is not motivated to end their alcohol use” (M-1949). The scholarly literature has also identified positive factors associated with older adults’ substance use, serving to fill in free time or to combat loneliness; for example, by going to a restaurant to meet others (Aubut et al., 2021).

Few clients were mentioned as misusing prescribed drugs or to simultaneously consume alcohol and prescription drugs, with some exceptions: “The client has used sedative drugs more than the doctor's orders and the drugs are running out …” (F-1933). At times, family members or relatives were documented as having given their own medications to the client with the aim of helping them: “The spouse said that they have for example given their own drugs to help the client sleep” (M-1942). This shows how substance use is often a relational activity that is not only used for socializing, but also as an attempt to help clients to live the everyday life they are used to and that they prefer. However, this may mean aggravating their substance use related problems and increasing their need for help and services.

Alongside present substance use, clients often had a documented history of heavy alcohol use. Some of the clients had needed hospital treatment because of alcohol-related issues, and others had formerly received treatment for substance use: “The client said that they had not been drinking alcohol recently, due to being afraid of falling. The client has fallen a few years ago because of intoxication and ended up in hospital” (F-1948). Different types of health issues were often described as underlying reasons for clients ending their use of alcohol: “(The client) has formerly used alcohol heavily and has now ended it because of health issues – nowadays small amounts (of alcohol) and rarely” (M-1944). The reasons for diminishing or ending alcohol use also related to accidents, fall-induced injuries, or to a fear of being injured, and the increased prevalence of injuries, falls, and cognitive and functional impairments have been connected to older adults’ substance use in numerous studies (Bye et al., 2021; Heuberger, 2009). In the following themes (II–IV) the point of view of the review is clients with ongoing substance use.

### Theme II: Complex needs for services

Older adults’ needs are often derived from a view of medicalization (Tiilikainen et al., 2025). The documentation of the clients’ needs for support focused on health and limitations in functional capacity, typically connected to the effects of ageing. In many cases, the assessment is performed by nurses (Kinni & Tiilikainen, 2024), which means that medicalization may be a result of professional emphasis. Sometimes these needs were used as the professionals’ justification for their client receiving services: “… The client might benefit from transfer to gerontological social work because of the client's age and capability of functioning” (M-1954). Some health conditions were described as unclear and requiring assessment, and clients were directed to healthcare services or to assessment and rehabilitation departments. Overall, clients were described as having several physical illnesses, injuries or difficulties in movement that required outpatient or inpatient care in hospital or other health or social care services: “The client ended up in the emergency department because of a fall. According to preliminary information, a friend had said that the client had been lying on the floor for two days” (F-1952). Low functional capability was often associated with dementia, which in some cases was related to alcohol use: “The client's functional capability and balance have gradually weakened because of (dementia) and alcohol use” (M-1942).

In addition to health-related issues, the clients had social challenges, which can be understood as typical for clients with complex needs (Ray et al., 2015; Soukiala & Pietilä, 2024). These challenges raised a need for support with daily activities and basic needs such as nutrition, hygiene, and securing medication: “The home care distributes the medication weekly and gives the medication daily to the client” (F-1947). Support was needed from domiciliary services with instrumental activities of daily living such as shopping or cleaning: “The apartment is untidy; the floors are sticky and there are flies” (F-1948). Besides taking care of living conditions, clients also needed support for financial issues (Rossi et al., 2025; Tiilikainen et al., 2025). The clients had challenges in managing their finances, or they were suspected of being victims of financial abuse, which then required the appointment of a public trustee. These challenges often affected the clients’ other needs, and their basic needs were often endangered: “The client is unable to manage their finances … Bills had not been paid, and the purchase of medicine and food was at risk” (M-1942).

A few clients had an acute need for housing support because they were left homeless after an eviction or due to having caused a disturbance. These situations required multiprofessional co-operation: “In cooperation with the assessment and rehabilitation unit, we need to consider how the housing of the client can be solved/whether it is possible to return to rental housing and where to get a rental apartment, if evicted and large foreclosures + safety risk/fire hazard in a case where a fire had been caused by the client themself, with the preliminary information being obtained from the property manager. The client cannot be left homeless” (F-1951). The pathway to homelessness may evolve over time (as with a long history of substance use), or it can take place rapidly; for example, when a spouse dies (Om et al., 2022). In some cases, the need for support was related to clients’ close relationships and the abuse that appeared in them. For example, domestic abuse was understood as creating a need for services: “The client is in need of special support due to physical abuse in the relationship” (M-1948). But at times, it was the violence of the client towards the partner that was used as a justification for service need, and, in these situations, the need for support focused on both the client and the partner.

The clients’ needs for support related to substance were rarely documented, which may be due to the challenges professionals’ face when addressing substance use (John Henley et al., 2018), or that the professionals did not present substance use as an issue justifying a need for support. Specifically, substance use related needs were often associated with acute situations related to intoxication, and these situations often involved other people and required help from several different services: “The client has repeatedly visited the casualty department and detoxification center. The police have visited the client's home nearly 30 times during this spring. […] The spouse has called for help because of the client's intoxication. The client has fallen several times and acted violently towards their spouse” (M-1949). The needs for more long-term support also related to illnesses or injuries which were caused by alcohol use: “The client used alcohol heavily during the summer, which is why the client was referred to hospital” (M-1954). In these cases, the clients’ needs were typically related to health issues (Tiilikainen et al., 2025).

### Theme III: Diverse network of care and support

Clients with ongoing substance abuse received care and support from both formal and informal networks. The clients were documented as using a wide range of social and healthcare and other services, which, in the data, features 27 different types of services. On average, each client had notes on using or being redirected to eight different kinds of services. Most service use was related to health and social care, with the most frequently used services being home care, healthcare centers, emergency units and hospitals. The clients’ everyday lives were also supported by various services, including guardians, intermediate account services, and shopping or meal delivery services (Rossi et al., 2025). The use of substance abuse services was rarely documented, and only one-third of clients with ongoing substance abuse had used services such as outpatient care, detoxification centers and rehabilitation in hospital, which may be affected by the scarcity of these kinds of services targeted towards older adults (Kuussaari et al., 2021). However, some of the clients had documentations related to substance abuse that had required police intervention at home, which were typically related to domestic violence or disturbances.

In addition to support from services, the clients received informal help from their families and kin, which typically play a significant role in long term care in Finland (Forma & Leinonen, 2024). This support and care varied from help with daily activities to comprehensive care for the client including, for example, household work, ensuring medication was taken, cooking and buying groceries. Family members also often took care of clients’ economic affairs, such as by helping to pay bills, and often arranged healthcare appointments. In the documents, the role of informal care was apparent in two ways and in some cases reduced the help needed from services. In some cases, the support from the family replaced the formal services: “No need for assigned caretaker, the spouse takes care of client's bank affairs, etc., with help of a letter of attorney” (F-1948), and, in some cases, the informal help was served to complement the formal support.

Nevertheless, at times, the family members’ active role in offering support had negative consequences for the clients. Family members were documented to have misused clients’ money, and their ability to help the client was sometimes limited: “The spouse had helped the client to pay bills in the online banking system. However, the spouse did not always have enough resources to take care of the client's economic affairs. Consequently, the bills had not been paid on time …” (F-1947). Sometimes, the family members actions turned to abusive behavior towards the client (Tiilikainen et al., 2025) and endangered the client's safety when they were unable to help: “The conditions in the home are challenging and unsafe. The spouse has prevented services from home care, and the client does not receive the help or care needed from the spouse at home” (M-1942). Substance abuse among older adults has been found to be a risk factor for becoming a victim of different kinds of abuse (Wadd & Galvani, 2014).

### Theme IV: Challenges in accessing social and healthcare services

Although clients were assisted by a wide range of services, some were described as outside the services, despite having been assessed as needing them. The absence from services was often caused by something that the clients did or did not do and focused on services not related to substance use. The challenges in accessing services were often related to clients refusing the services that were offered: “The client refuses to accept cleaning services, and has instead been cleaning by themself when prompted … The client does not want to use the shopping service as it does not allow purchasing cigarettes …” (M-1946). Some of these clients were described as having an ambivalent attitude towards receiving help from services, which challenged their clientship in the services. Their redirection to services might also have been a long process, where clients might occasionally accept help from the services, and then at other times for example refuse to let the professionals into their homes, fail to go to appointments, or interrupt their inpatient care. Balancing between a client's unwillingness to receive services and respecting their right to self-determination is one of the challenges that professionals encounter when offering services to clients, and this has also been related to older clients’ substance abuse (Peiponen et al., 2023). In some cases, the clients were unable to seek help which related to their substance abuse: “The home care comes three times a day and the client might be at the nearby bar, which means that medicines are then not taken” (F-1947). In these situations, intoxication might have been a barrier to receiving help, or the home care routines did not match the preferred timetables of the client (Jönson et al., 2024).

Being outside substance abuse services was often related to the client's lack of motivation to reduce or stop their substance use, or to seek help through substance abuse treatment. In these situations, the social and healthcare professionals had often been concerned about the client's alcohol use for a long period of time. These situations were often preceded by professionals’ persistent work and dialogue with their clients: “The client has a history of long-lasting alcohol abuse. The client has sometimes been sober for a long time but has then started to drink again. The client has been offered help with their substance abuse problem but is not motivated to participate in rehabilitation” (M-1946). In some cases, clients did not experience their alcohol use as being problematic despite the expressed concern of professionals, and did not see any need to seek help. These situations reflect dilemmas that are typical in the welfare sector, when professionals who are working with older adults with substance abuse must balance between clients’ autonomy and simultaneously provide care which is shaped by guidelines and the professional's training and experience (Bjerge et al., 2024).

## Discussion

There was a continuum on how substance use was described in client documents, ranging from not using, to not detecting use, to non-harmful use, and ending with harmful use. This observation is in line with the prevailing understanding of the spectrum of alcohol use (Cohen et al., 2022). The use of illegal drugs was not present, but the misuse of prescription drugs or the use of them in conjunction with alcohol were mentioned. Only a small number of clients were identified as using substances harmfully and in manners that affected their life situations. The client service needs from services were assessed through client documents, which revealed complex life situations involving entwined health issues, functional decline, social problems and economic challenges (Ray et al., 2015; Rossi et al., 2025; Soukiala & Pietilä, 2024) and portrayed the intricate web of actors that are present and needed when working with this group of clients.

However, the clients’ service use and needs related primarily to health issues and were rarely documented from the point of view of substance use, even though problematic substance use had been included as a criterion of having a status of complex service needs (Kuusisto et al., 2021). Drawing from the documentation, it seems possible that gerontological social work clients’ substance use related needs are not recognized at an adequate level in the assessment process. It is also noteworthy that only a minority of clients with ongoing substance abuse were documented as using substance abuse services, which may reflect the scarcity of resources in substance abuse services targeted towards older adults (Peiponen et al., 2023; Wadd & Dutton, 2018).

Another significant observation was that the professionals tend to have ignored substance use when assessing the needs of older clients. The data show that, in a significant number of cases, the professionals either did not discuss substance use with their clients or they have had challenges in identifying substance use. These results are congruent with previous studies (Bareham et al., 2021; Johannessen et al., 2021; Wadd & Galvani, 2014; Weber et al., 2022). On the other hand, it has been noted that health and social care professionals may interpret symptoms such as hypertension, falls, depression, self-harm and diabetes as being age-related, rather than reflecting problematic substance use (Karlsson & Gunnarsson, 2018). This can be interpreted from the point of view of medicalization of ageing whereby ageing processes and older adults are increasingly subjected to society's biomedical scrutiny resulting in misinterpretation of substance abuse (Jønsson, 2024).

The difficulties in identifying substance abuse might also be related to insufficient training and the working methods of social and healthcare professionals (Bareham et al., 2021; Bjerge et al., 2024; Karlsson & Gunnarsson, 2018) encountering older clients. Overall, social workers should have the basic knowledge and skills to identify and understand substance abuse and related problems (McCarthy & Galvani, 2004). Failures in recognizing substance abuse increases the risk of excluding older adults from the service system (Peiponen et al., 2023). This is a problem as substance abuse services targeted towards older adults are limited (Kuussaari et al., 2021; Wadd & Dutton, 2018) and older adults who use substances harmfully are often excluded from the formal substance abuse service system (Peiponen et al., 2023; Quinn, 2020).

The present study has some limitations. The study focused on one municipality in Finland, and gerontological social work methods and documentation practices may vary between regions. Therefore, to gain a more comprehensive view, there is a need for wider research across Finland. It is also acknowledged that, while this study researched the professionals’ point of view in the documentation, the research lacks the client perspective (Kuorikoski, 2024). To strengthen the clients’ position, there is a need for further research which considers the clients’ view in the documentation and assessment process.

According to legislation, clients with substance abuse need special support, but, in the case of older adults, these needs are often not identified. Our results emphasize a need to better train social workers to identify, document and discuss substance use when encountering older clients. This kind of training could be offered for example by open universities.

Our results indicate that older adults with substance abuse use a wide range of services within the social and healthcare system, but the focus in these services is not on substance abuse. Despite not being substance abuse services, these services are essential in identifying substance abuse and steering the clients to substance abuse services. At present, for example, long-term care services tend to be an ill fit with older adults who have both care needs and substance abuse issues (Bach et al., 2024; Koivula et al., 2016). At the same time, social and healthcare services that are targeted towards older adults should be developed to appropriately answer the increase of substance abuse of older adults and to recognize the complex needs of older clients with substance abuse issues. Because of the increasing numbers of older adults, it is essential to place substance use and abuse more centrally in the social and healthcare system and in the education and research of gerontological social work.

## Notes

1. The concept substance abuse is here chosen due to the Finnish terminology used in the research.

2. We refer to the clients with gender female (F) or male (M) and birth year at the end of data excerpts.
